# Supplementary material for: Brain functional connectivity, but not neuroanatomy, captures the interrelationship between sex and gender in preadolescents
Source: Dev Cogn Neurosci. 2025 Oct 3;76:101624. doi: 10.1016/j.dcn.2025.101624 (PMC12539272; doi:10.1016/j.dcn.2025.101624)
Supplement: Supplementary file 1 — Supplementary material [file mmc1.docx]

**Supplementary Materials**

# **Supplementary Methods**

## *Support Vector Machine Classification & Regression*

The MATLAB (The MathWorks Inc., 2023) fitclinear function was used to construct the univariate and multivariate models that classified participants as male or female. Here, the notation (“option name,” “option choice”) is used to describe the choices made when using the fitclinear function. Support vector machine (SVM) (“Learner,” “svm”) was chosen for the binary classification model. Regularization was performed with the ridge (L2-norm) penalty (“Regularization,” “ridge”). Dual stochastic gradient descent for SVM (“Solver,” “dual”) was the objective function minimization technique. Lastly, grid search optimization of the regularization coefficient C was performed. This optimization attempts to minimize the cross-validation loss (error) determining the balance between the training errors and the generalizability of the SVM classification model. Specifically, it searches for the optimum among a given C range. In this case, the range of C was [2^-10^, 2^-8^, …, 2^12^, 2^14^] (i.e., 13 values in total) (Hsu et al., 2003). Inner accuracies were calculated for each C value and the C with the highest mean inner prediction accuracy was chosen as the optimal C (Cui & Gong, 2018; Hsu et al., 2003) for testing in each of the outer fold test sets. The support vector machine regression (SVR) models were constructed using MATLAB’s fitrlinear function, with the same parameters and choices as those applied in the SVM classification models.

- 1. *Validation Analyses*

As a form of validation, we trained rsFC, cortical thickness, cortical volume, rsFC/cortical thickness, and rsFC/cortical volume SVM models on unaligned participants and tested them on unaligned and aligned participants (Supplementary Fig. 2).

# **Supplementary Results**

## *Cortical thickness SVM classification at the vertex-level*

We performed SVM classification to examine the predictability of sex in adolescents. We used cortical thickness at the vertex-level (59,412 cortical vertices for each participant) in an effort to capture regional effects that may not align with ROI boundaries. The cortical thickness vertex-level sex classifier was trained on the same aligned participants as the rsFC and ROI-level cortical thickness classifiers. It correctly separated aligned males from females with an accuracy of 81% (*p* < 0.001). Sensitivity, specificity, AUC, and MCC of the model were 0.80, 0.83, 0.87, and 0.63 respectively. Variation in the anatomical organization of cortical vertices belonging to the medial visual, visual, premotor, somatomotor mouth, and frontoparietal networks, in that order, contributed the most to the model and were therefore relatively more important in predicting participant sex. The rsFC sex classifier performed numerically better than the vertex-level cortical thickness sex classifier (85% vs 81%), which in turn performed numerically better than the ROI-level cortical thickness sex classifier (81% vs 76%). However, neither performance difference reached statistical significance (McNemar’s tests: rsFC vs. vertex-level cortical thickness: *χ²* = 1.24, *p* = 0.27; vertex-level vs. ROI-level cortical thickness: *χ²* = 2.34, *p* = 0.13). These findings highlight the superior performance of the rsFC classifier in predicting sex while emphasizing the limitations of the cortical thickness classifiers. The fact that the vertex-level cortical thickness classifier surpassed the ROI-level classifier’s performance implies that maintaining higher spatial resolution in cortical thickness data may enhance its effectiveness for sex classification.

Next, we assessed the classifier’s efficacy in predicting the sex of individuals with sex/gender unalignment. The vertex-level cortical thickness sex classifier trained on the aligned participants was able to classify unseen participants with sex/gender unalignment as male or female with 76% accuracy (*p <* 0.001; Sensitivity, specificity, AUC, MCC = 0.85, 0.72, 0.87, and 0.54). The vertex-level cortical thickness SVM model predicting the independent aligned group achieved statistically significantly higher prediction accuracy (81%) compared to the unaligned group (76%; *z* = 2.12, *p* = 0.03).

The vertex-level cortical thickness sex classifier did not show significantly better performance in predicting unaligned individuals compared to the rsFC classifier (McNemar’s test: *χ^2^* = 0.57, *p =* 0.45). It did however show significantly better performance compared to the ROI-level cortical thickness classifier (*χ^2^* = 4.47, *p* = 0.03).

Lastly, we investigated the relationship between the vertex-level cortical thickness scores (brain profile predictions) and sex/gender youth-self reported and parent-reported sex/gender alignment scores to evaluate the consistency between the adolescents’ brain profiles and sex/gender alignment. The correlations between vertex-level cortical thickness classification and sex/gender alignment scores were not significant for females (*ρ* = 0.06, *p* = 0.08), but were significant for males (*ρ* = -0.11, *p* < 0.01) exhibiting the same inverse relationship as in the rsFC and ROI-level cortical thickness patterns.

## *Cortical thickness SVR at the vertex-level*

Following the SVM classification, we assessed whether an adolescent’s degree of sex/gender alignment can be predicted by vertex-level cortical thickness. The SVM classification was extended to examine linear patterns among features that predict a continuous variable (here, sex/gender alignment as assessed by the Youth Self-Report and Parent-Report Gender Questionnaires) with linear SVR. Using the same parameters/choices as in the SVM classification models, we ran a total of four models, utilizing different combinations of features and questionnaire types: One model exclusive to females and one exclusive to males, each utilizing the Youth Self-Report Gender Questionnaire as the target variable in one iteration and the Parent-Report Gender Questionnaire in another. Similar to the rsFC and ROI-level cortical thickness SVR models, neither vertex-level sex/gender alignment SVR model successfully predicted the sex/gender alignment Youth Self-Report and Parent-Report Gender Questionnaire scores. Correlations between the original sex/gender alignment scores and the predicted sex/gender alignment scores were not significant (all *p*-values > .05), with the exception of the correlation between original parent-reported sex/gender alignment scores and predicted scores in males (p = 0.02). However, all coefficients of determination were negative (R² < 0), including the exception (*R²* = -0.41). This suggests that the models performed worse than a null model that predicts the mean of the dependent variable (sex/gender alignment scores) for all observations.

- 1. *Validation analyses*
     1. *Functional connectivity and neuroanatomical classification of sex*

The rsFC sex classifier trained on the unaligned participants was able to classify unseen unaligned participants from the independent testing set as male or female with 74% accuracy (*p* < 0.001). Sensitivity, specificity, AUC, and MCC of the model were 0.78, 0.72, 0.81, and 0.49 respectively. Variations in the functional organization of the visual, action mode, medial visual, default mode, and auditory networks, in that order, contributed the most to the model and were therefore relatively more important in predicting participant sex.

The cortical thickness sex classifier was trained on the same unaligned participants as the rsFC classifier. It correctly separated unaligned males from females with an accuracy of 76% (*p* < 0.001). Sensitivity, specificity, AUC, and MCC of the model were 0.74, 0.77, 0.79, and 0.50 respectively. Variation in the anatomical organization of cortical parcels belonging to the visual (medial visual and visual), frontoparietal, action mode, and default mode networks, in that order, contributed the most to the model and were therefore relatively more important in predicting participant sex.

The cortical volume sex classifier correctly separated unaligned males from females with an accuracy of 70% (*p* < 0.001). Sensitivity, specificity, AUC, and MCC of the model were 0.76, 0.68, 0.79, and 0.41 respectively. Variation in the anatomical organization of cortical parcels belonging to the language, frontoparietal, visual, action mode, and dorsal networks, in that order, contributed the most to the model and were therefore relatively more important in predicting participant sex.

The combined rsFC/cortical thickness sex classifier correctly separated unaligned males from females with an accuracy of 74% (*p* < 0.001). Sensitivity, specificity, AUC, and MCC of the model were 0.81, 0.71, 0.84, and 0.49 respectively. Variations in the organization of the visual, action-mode, default mode, dorsal attention, and medial visual networks, in that order, contributed the most to the model and were therefore relatively more important in predicting participant sex.

Lastly, the combined rsFC/cortical volume sex classifier correctly separated unaligned males from females with an accuracy of 74% (*p* < 0.001). Sensitivity, specificity, AUC, and MCC of the model were 0.81, 0.71, 0.84, and 0.49 respectively. Variations in the organization of the visual, default mode, action-mode, dorsal attention, and medial visual networks, in that order, contributed the most to the model and were therefore relatively more important in predicting participant sex.

The rsFC and cortical thickness sex classifiers performed equally well in predicting unaligned individuals (74% vs 76%) with no significant difference in accuracy (McNemar’s test: *χ^2^* = 0.08, *p* = 0.77). Although the rsFC sex classifier achieved numerically higher accuracy (74%) compared to the cortical volume classifier (65%), that difference did not reach statistical significance (McNemar’s test: *χ^2^* = 2.69, *p =* 0.10). Yet there was a significant difference between the performance of the cortical thickness (76%) and cortical volume (65%) sex classifiers (McNemar’s test: *χ^2^* = 4.07, *p =* 0.04). The combined rsFC/cortical thickness and rsFC cortical volume sex classifiers performed equally well as the rsFC classifier (McNemar’s tests: *χ^2^* = 2.96, *p* = 0.09 and *χ^2^* = 0.01, *p* = 0.93) but only the rsFC/cortical thickness sex classifier performed significantly better than the cortical thickness one (McNemar’s test: *χ^2^* = 4.40, *p =* 0.04). The cortical volume sex classifier did not perform significantly better compared to the rsFC/cortical volume one (McNemar’s test: *χ^2^* = 2.69, *p* = 0.10). Lastly, the combined rsFC/cortical thickness sex classifier did not perform significantly better than the combined rsFC/cortical volume sex classifier (McNemar’s test: *χ^2^* = 2.96, *p* = 0.09).

## *Sex classifiers’ efficacy in predicting aligned and unaligned individuals*

The rsFC sex classifier trained on the unaligned participants was able to classify unseen aligned participants as male or female with 72% accuracy (*p <* 0.001). Sensitivity, specificity, AUC, and MCC of the model were 0.72, 0.73, 0.79, and 0.45 respectively. The rsFC SVM model trained in unaligned individuals and predicting the independent unaligned group achieved numerically higher prediction accuracy (74%) than predicting the aligned group (72%; *z* = -0.66, *p* = 0.53).

The cortical thickness sex classifier trained on the unaligned participants was able to classify unseen aligned participants as male or female with 69% accuracy (*p <* 0.001; Sensitivity, specificity, AUC, MCC = 0.62, 0.79, 0.78, and 0.41). The cortical thickness SVM model did not achieve a statistically significantly higher prediction accuracy for the unaligned independent testing set (76%) compared to the aligned group (69%; *z* = -2.14, *p* = 0.03).

The cortical volume sex classifier was able to classify unseen aligned participants as male or female with 69% accuracy (*p <* 0.001; Sensitivity, specificity, AUC, MCC = 0.64, 0.76, 0.77, and 0.40). The cortical volume SVM model did not achieve a statistically significantly higher prediction accuracy for the unaligned independent testing set (70%) compared to the aligned group (69%; *z* = 0.37, *p* = 0.71).

The combined rsFC/cortical thickness classifier sex classifier was able to classify unseen aligned participants as male or female with 66% accuracy (*p <* 0.001; Sensitivity, specificity, AUC, MCC = 0.65, 0.66, 0.74, and 0.31). The rsFC/cortical thickness SVM model did not achieve a statistically significantly higher prediction accuracy for the unaligned independent testing set (65%) compared to the aligned group (66%; *z* = 0.21, *p* = 0.83).

The combined rsFC/cortical volume classifier sex classifier was able to classify unseen aligned participants as male or female with 72% accuracy (*p <* 0.001; Sensitivity, specificity, AUC, MCC = 0.72, 0.73, 0.79, and 0.44). The rsFC/cortical volume SVM model did not achieve a statistically significantly higher prediction accuracy for the unaligned independent testing set (74%) compared to the aligned group (72%; *z* = -0.69, *p* = 0.49).

The rsFC sex classifier trained in unaligned individuals performed equally well in predicting sex for aligned individuals as the cortical thickness (McNemar’s test: *χ^2^* = 3.39, *p* = 0.07) and the cortical volume classifiers (McNemar’s test: *χ^2^* = 3.60, *p* = 0.06). The cortical thickness and cortical volume sex classifiers also performed equally well (McNemar’s test: *χ^2^* < 0.001, *p* = 0.98). The rsFC sex classifier performed significantly better than the rsFC/cortical thickness one (McNemar’s test: *χ^2^* = 14.65, *p* < 0.001) but equally well as the rsFC/cortical volume one (McNemar’s test: *χ^2^* = 0.01, *p* = 0.93). Neither cortical thickness nor cortical volume performed significantly better compared to the rsFC/cortical thickness sex classifier (McNemar’s test: *χ^2^* = 3.85, *p* = 0.05) or the cortical volume classifier (McNemar’s test: *χ^2^* = 3.16, *p* = 0.08) respectively. Lastly, the combined rsFC/cortical volume sex classifier performed significantly better than the combined rsFC/cortical thickness sex classifier (McNemar’s test: *χ^2^* = 13.75, *p* < 0.001).

Comparative analyses of SVM models trained (and tested) on individuals with sex/gender alignment versus those trained (and tested) on unaligned individuals (validation SVMs) revealed that the aligned SVM models achieved significantly higher performance when predicting aligned individuals using rsFC (*z* = 3.19, *p* < 0.01), rsFC/cortical thickness (*z* = 5.58, *p* < 0.001), and rsFC/volume (*z* = 2.93, *p* < 0.01) features. In contrast, when relying solely on cortical thickness (*z* = -0.01, *p* = 0.99) or volume metrics (*z* = -0.18, *p* = 0.86), no statistically significant differences in predictive accuracy emerged between the aligned and unaligned (validation) SVMs.

- - 1. *Discussion*

The validation analyses corroborate the main findings, with a consistent pattern of results emerging across both sets of analyses. Notably, when examining the SVM classifiers trained and tested in unaligned participants, the rsFC-based model did not achieve statistically significant performance over the cortical thickness and cortical volume models. Furthermore, the rsFC-based classifiers trained and tested in aligned individuals demonstrated significantly superior accuracy compared to their unaligned counterparts. Both of these outcomes likely reflect the advantage of using labels from a more homogeneous subpopulation with respect to sex/gender alignment (SVM classifiers trained using only aligned individuals), which reduces label noise and allows the classifier to more accurately capture the distinctions between sexes. Establishing this baseline performance with optimized label quality provides a benchmark for evaluating classifier efficacy under more heterogeneous conditions. Additionally, the smaller sample size in the unaligned training group (*n* = 893 vs *n* = 1,610 aligned training group; Supplementary Fig. 2) likely reduced statistical power, potentially limiting the ability to detect significant differences in performance across models.

# **Supplementary Figures**

**Supplementary Figure 1.** Frequency distribution of the (A) Youth Self-Report and (B) Parent-Report Gender Questionnaires.

**Supplementary Figure 2. Flowchart of the support vector machine (SVM) validation model construction.** For the validation analyses, the dataset was split in two groups: Aligned (participants with sex/gender alignment) and unaligned (participants with sex/gender unalignment). The unaligned group was split in an aligned training group (80%) and an unaligned hold-out testing group (20%). A nested five-fold cross-validation (5F-CV) was employed, with the inner 5F-CV determining the optimal parameter C and the outer 5F-CV estimating the generalizability of the model. The final, optimal model was subsequently applied in the held-out unaligned testing group and the aligned group, and model performance was evaluated. The forward slash in the groupings denotes the different possible group splits (e.g. The performance estimation loop training group was split in three train/test group pairs with 714/179 subjects and two train/test group pairs with 715/178 subjects respectively).

# **Supplementary Tables**

**Supplementary Table 1. ABCD Gender Survey Questions.**

| **Survey** | **Item** | **Construct** |
| --- | --- | --- |
| Youth Self-Report Gender Identity Questionnaire | How much do you feel like a <boy/girl>? | Sex-congruent felt-gender |
|  | How much do you feel like a <girl/boy>? | Sex-incongruent felt-gender |
|  | How much have you had the wish to be a <girl/boy>? | Gender non-contentedness |
|  | How much have you dressed or acted as a <girl/boy> during play? | Gender non-conformity |
| Parent-Report Gender Identity Questionnaire | <His/Her> favorite playmates are: | Sex-typed behavior during play |
|  | <He/She> plays with girl-type dolls, such as “Barbie”. |  |
|  | <He/She> plays with boy-type dolls such as action figures or “GI-Joe”. |  |
|  | <He/She> experiments with cosmetics (makeup) and jewelry. |  |
|  | <He/She> imitates female characters seen on TV or in the movies. |  |
|  | <He/She> imitates male characters seen on TV or in the movies. |  |
|  | <He/She> plays sports with boys (but not girls). |  |
|  | <He/She> plays sports with girls (but not boys). |  |
|  | *In playing “mother/father”, “house”, or “school” games, <he/she> takes the role of:* |  |
|  | <He/She> plays “girl/boy-type” games (as compared to “boy/girl-type” games). |  |
|  | *In dress-up games, <he/she> likes to dress up as:* |  |
|  | <He/She> states the wish to be a <girl/boy> or <woman/man>. | Gender dysphoria |
|  | <He/She> states that <he/she> is a <girl/boy> or <woman/man>. |  |
|  | <He/She> talks about not liking <his/her> sexual anatomy (private parts). |  |

**Supplementary Table 2. Family income and descriptive statistics.**

|  | TOTAL (n = 3,129) | |  |
| --- | --- | --- | --- |
|  | Males  n = 1,542; 49.3% | Females  n = 1,587; 50.7% |  |
| Family Income |  |  |  |
| < $5,000 | 31 (2%) | 25 (1.6%) | χ^2^ = 0.84, p = 0.36 |
| $5,000 – $11,999 | 27 (1.8%) | 34 (2.1%) | χ^2^ = 0.63, p = 0.43 |
| $12,000 – $15,999 | 26 (1.7%) | 20 (1.6%) | χ^2^ = 0.98, p = 0.32 |
| $16,000 – $24,999 | 50 (3.2%) | 50 (3.2%) | χ^2^ = 0.02, p = 0.88 |
| $25,000 – $34,999 | 82 (5.3%) | 77 (4.9%) | χ^2^ = 0.35, p = 0.55 |
| $35,000 – $49,999 | 97 (6.3%) | 104 (6.6%) | χ^2^ = 0.09, p = 0.76 |
| $50,000 – $74,999 | 181 (11.7%) | 190 (12%) | χ^2^ = 0.04, p = 0.84 |
| $75,000 – $99,999 | 210 (13.6%) | 218 (13.7%) | χ^2^ < 0.01, p = 0.92 |
| $100,000 – $200,000 | 520 (33.7%) | 566 (35.7%) | χ^2^ = 1.30, p = 0.25 |
| > $200,000 | 220 (14.3%) | 215 (13.5%) | χ^2^ = 0.34, p = 0.56 |

**Supplementary Table 3.** **Demographics (n = 3,129) and descriptive statistics for aligned and unaligned groups.** SD, standard deviation; PDS, pubertal development scale; FD framewise displacement.

|  | TOTAL (n = 3,129) | |  |
| --- | --- | --- | --- |
|  | Aligned  n = 2,013; 64.3% | Unaligned  n = 1,116; 35.7% |  |
| Mean Age in Months (SD)  Range | 132.6 (7.7)  116-149 | 131.9 (7.8)  117-147 | ***t = 2.46, p = 0.01*** |
| Sex |  |  |  |
| Male | 1,153 (57.3%) | 389 (34.9%) | ***χ^2^ = 144.40, p < 0.001*** |
| Female | 860 (42.7%) | 727 (65.1%) |  |
| PDS |  |  |  |
| Prepuberty | 514 (25.5%) | 179 (16%) | ***χ^2^ = 37.54, p < 0.001*** |
| Early puberty | 704 (35%) | 339 (30.4%) | ***χ^2^ = 6.83, p < 0.01*** |
| Mid puberty | 700 (34.8%) | 496 (44.4%) | ***χ^2^ = 28.43, p < 0.001*** |
| Late puberty | 95 (4.7%) | 102 (9.1%) | ***χ^2^ = 23.78, p < 0.001*** |
| Residual In-Scanner Motion (Mean FD), mm | 0.12 (0.07)  0.03-0.66 | 0.12 (0.09)  0.03-1.07 | t = -0.81, p = 0.42 |

**Supplementary Table 4. Family income and descriptive statistics for aligned and unaligned groups.**

|  | TOTAL (n = 3,129) | |  |
| --- | --- | --- | --- |
|  | Aligned  n = 2,013; 64.3% | Unaligned  n = 1,116; 35.7% |  |
| Family Income |  |  |  |
| < $5,000 | 41 (2%) | 15 (1.3%) | χ^2^ = 1.96, p = 0.16 |
| $5,000 – $11,999 | 39 (2%) | 22 (2%) | χ^2^ < 0.01, p = 0.95 |
| $12,000 – $15,999 | 34 (1.7%) | 12 (1.1%) | χ^2^ = 1.87, p = 0.17 |
| $16,000 – $24,999 | 60 (3%) | 40 (3.6%) | χ^2^ = 0.84, p = 0.36 |
| $25,000 – $34,999 | 104 (5.2%) | 55 (5%) | χ^2^ = 0.08, p = 0.77 |
| $35,000 – $49,999 | 122 (6.1%) | 79 (7.1%) | χ^2^ = 1.24, p = 0.27 |
| $50,000 – $74,999 | 226 (11.2%) | 145 (13%) | χ^2^ = 2.14, p = 0.14 |
| $75,000 – $99,999 | 259 (12.9%) | 169 (15.1%) | χ^2^ = 3.15, p = 0.08 |
| $100,000 – $200,000 | 711 (35.3%) | 375 (33.6%) | χ^2^ = 0.94, p = 0.33 |
| > $200,000 | 294 (14.6%) | 141 (12.6%) | χ^2^ = 2.33, p = 0.13 |

# **Supplementary Equations**

$$MCC= \frac{Cov(c,l)}{\sigma_{c}*\sigma_{l}}= \frac{TP*TN-FP*FN}{\sqrt{\left( TP+PF \right)*\left( TP+FN \right)*\left( TN+FP \right)*(TN+FN)}}$$

**Supplementary Equation 1. Matthews Correlation Coefficient.** Measures the correlation of the true classes *c* with the predicted labels *l*. Worst value = -1; Best value = +1. *Cov*(*c*,*l*): covariance of the true classes *c* and predicted labels *l*; *σ_c_*: standard deviation of the true classes; *σ_l_*: standard deviation of the predicted labels; TP: True Positives; TN: True Negatives; FP: False Positives; FN: False Negatives.

# **References**

Cui, Z., & Gong, G. (2018). The effect of machine learning regression algorithms and sample size on individualized behavioral prediction with functional connectivity features. *Neuroimage*, *178*, 622–637. https://doi.org/10.1016/j.neuroimage.2018.06.001

Hsu, C.-W., Chang, C.-C., & Lin, C.-J. (2003). *A Practical Guide to Support Vector Classification*. http://www.csie.ntu.edu.tw/~cjlin

The MathWorks Inc. (2023). *MATLAB* (9.14.0 (R2023a)). The MathWorks Inc. https://www.mathworks.com
